# Topical Treatment of Eyebrow Hypotrichosis with Bimatoprost 0.03% Solution: Case Report and Literature Review

**DOI:** 10.7759/cureus.2666

**Published:** 2018-05-21

**Authors:** Ryan R Riahi, Philip R Cohen

**Affiliations:** 1 Dermatology, Derm Surgery Associates, PA; 2 Department of Dermatology, University of California, San Diego, San Diego, USA

**Keywords:** alopecia, bimatoprost, eyebrow, growth, hair, hypotrichosis, madarosis, prostamide, therapeutic, therapy

## Abstract

The eyebrows frame the upper margin of the orbit and are an essential feature of the facial landscape. Eyebrow hypotrichosis, also known as madarosis, is characterized by a lack of growth or loss of eyebrow hair. Eyebrow loss can have cosmetic, functional, and social consequences. Eyebrow hypotrichosis can be idiopathic or related to an underlying condition. Bimatoprost 0.03% solution is a prostamide F_2α_ analog indicated for the treatment of glaucoma and ocular hypertension that has also demonstrated efficacy for hair growth; indeed, it is currently approved by the Food and Drug Administration for the treatment of eyelash hypotrichosis. A 60-year-old woman with eyebrow hypotrichosis is described who achieved excellent and sustained growth of her eyebrows with continual daily application of bimatoprost 0.03% solution. We discuss the therapeutic mechanisms of bimatoprost 0.03% solution in hair growth, review other potential modalities for treating eyebrow hypotrichosis, and summarize the findings of investigators who have utilized bimatoprost in the treatment of eyebrow hypotrichosis.

## Introduction

Eyebrows serve an important role in communication and cosmetic appearance. Eyebrow hypotrichosis is characterized by the lack of growth or loss of eyebrow hair [1]. This condition can be idiopathic or related to an underlying disorder [1]. Bimatoprost 0.03% ophthalmic solution is a prostamide F_2α_ analog approved for the treatment of glaucoma and ocular hypertension that has also demonstrated efficacy in hair growth [2-9]. We describe a 60-year-old woman with eyebrow hypotrichosis who achieved excellent and sustained growth of her eyebrows with continual daily application of bimatoprost 0.03% solution.

## Case presentation

A 60-year-old healthy Caucasian woman presented for evaluation and treatment for eyebrow alopecia; she did not have any other site of hair loss. She reported having thin eyebrows and would previously shape her eyebrows by plucking the hairs with tweezers. She did not have any other medical conditions.

**Figure 1:**
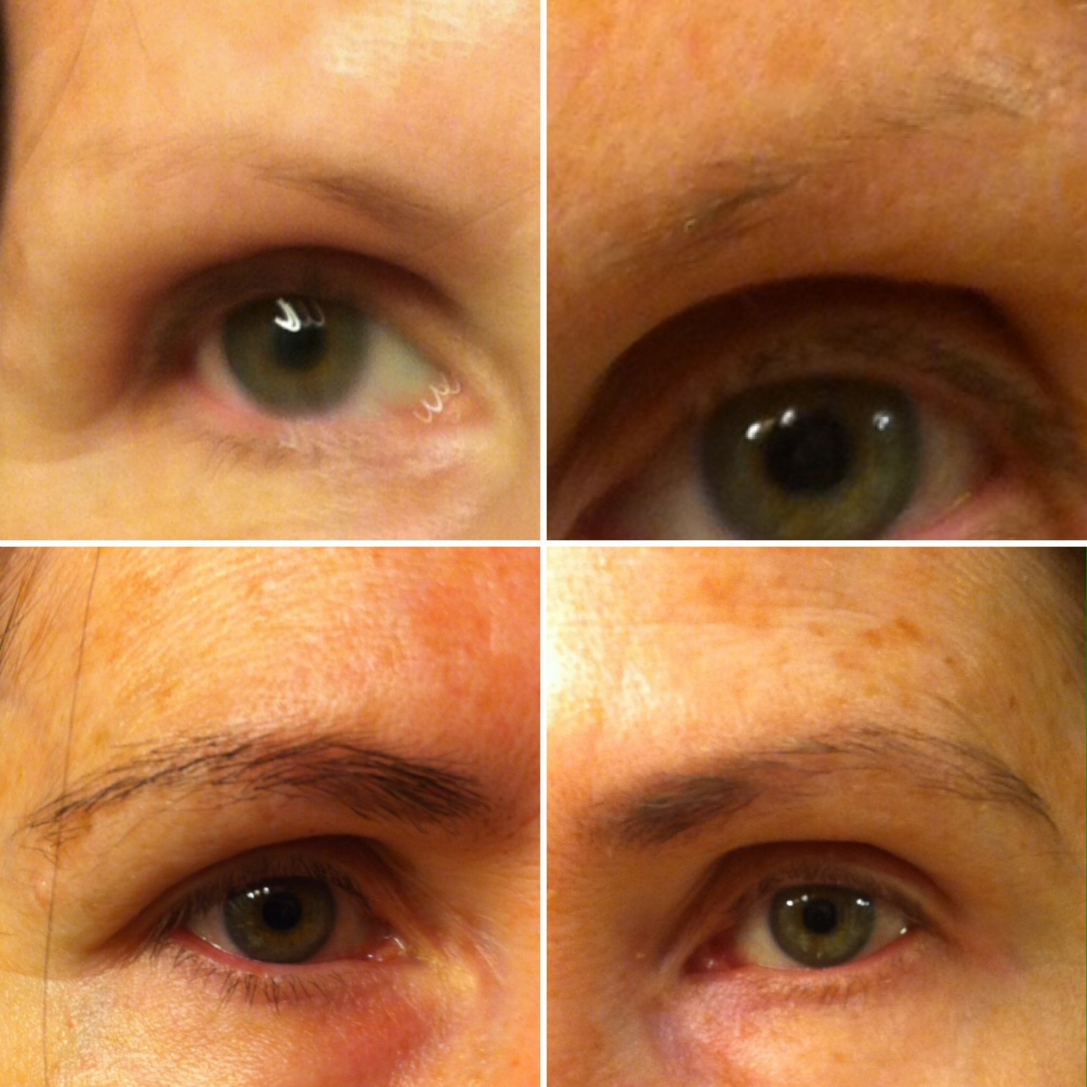
Topical bimatoprost 0.03% solution treatment of eyebrow hypotrichosis The right and left eyebrows of a 60-year-old woman before topical bimatoprost 0.03% solution treatment (top row) and after eight months of once daily application of bimatoprost 0.03% solution (bottom row).

Examination of the eyebrows revealed sparse and thin black hairs (Figure 1). Examination of her scalp and face did not reveal alopecia elsewhere; specifically, neither frontal hairline recession nor temporal cobble stoning were noted. Similarly, she had no additional areas of hair loss on her body.

The patient was diagnosed with idiopathic eyebrow hypotrichosis. She was prescribed bimatoprost 0.03% solution for use to the affected areas daily. The patient was educated that improvement in her eyebrow hypotrichosis would be gradual. Periodic follow-up every two months was performed. At each visit, the patient reported compliance with once a day application of the bimatoprost 0.03% solution; increased hair growth and thickening of the eyebrow hairs was observed. She had no treatment-associated side effects. After eight months, she had complete regrowth of her eyebrows (Figure 1); her daily topical treatment with bimatoprost 0.03% solution is being continued.

## Discussion

The eyebrows frame the upper margin of the orbit and are an essential feature of the facial landscape. They serve not only a role in protecting the eyes, but also as a means of as non-verbal communication. Eyebrow loss can have cosmetic, functional, and social consequences [2]. Loss of eyebrow hair can be idiopathic or age-related. However, eyebrow hypotrichosis can be due to a local or systemic condition (Table 1) [1,2].

**Table 1:** Local or systemic conditions associated with eyebrow hypotrichosis

Associated condition	Examples
Autoimmune	Alopecia areata, discoid lupus erythematosus, frontal fibrosing alopecia
Dermatitis	Atopic dermatitis, psoriasis, seborrheic dermatitis
Endocrine	Thyroid disease (hyperthyroidism or hypothyroidism)
Exogenous	Radiation
Genodermatoses	Ectodermal dysplasias, Netherton syndrome
Infectious	Leprosy, syphilis (secondary)
Medications	Acitretin, chemotherapy, thallium, valproic acid
Neoplastic	Basal cell carcinoma, folliculotropic mycosis fungoides, squamous cell carcinoma
Nutritional disorder	Biotin deficiency, zinc deficiency
Systemic disorder	Amyloidosis, sarcoidosis
Trauma	Chemical burn, trichotillomania

Eyebrow hypotrichosis can be a cosmetic concern and impact the affected individual’s self-esteem [2]. Several treatments to benefit patients with eyebrow hypotrichosis have been explored. Available options include bimatoprost, hair transplantation, and minoxidil [Table 2] [2-12].

**Table 2:** Medical and surgical treatments for eyebrow hypotrichosis

Treatment	Reference
Autologous fat grafting	[12]
Bimatoprost (topical)	[2-9, current report]
Hair transplantation	[11]
Minoxidil (topical)	[6, 10]

Bimatoprost was approved by the Food and Drug Administration for the treatment of eyelash hypotrichosis in 2008 [9]. The medication is a prostamide F_2α_ analogue; it demonstrated the side effect of eyelash growth when utilized in the treatment of glaucoma and ocular hypertension [2]. Ophthalmic use of bimatoprost also demonstrated side effects such as periorbital hyperpigmentation and iris pigmentation [2,13]. Darkening, as well as increased length and thickness of hair, is noted with continual use of bimatoprost 0.03% solution [13]. However, bimatoprost 0.03% solution does not increase the number of eyelash follicles [13].

The mechanism of action of bimatoprost 0.03% solution in the treatment of eyebrow hypotrichosis has been evaluated [6,13,14]. Bimatoprost exerts its effects by stimulating the prostamide receptor, leading to downstream effects resulting in the transition of hair follicles from the telogen phase to the anagen phase [6,13]. Bimatoprost treatment has also been associated with prolonging the duration of the anagen phase, leading to longer hair [13]. Investigators have postulated that the clinical improvement in eyelash fullness and thickness that has been observed is due to enlargement of the dermal papillae and an increase in hair bulb diameter [13]. In addition, researchers have attributed the mechanism of hair darkening noted with bimatoprost use to result from increased melanogenesis via stimulation of the enzyme tyrosinase [13,14].

Prompted by the excellent response of eyelash growth with use of bimatoprost 0.03% solution, a small number of investigators have utilized bimatoprost to treat eyebrow hypotrichosis (Table 3) [3-7,9]. The studies included both women and men. Including our patient, a total of 421 patients have been evaluated.

**Table 3:** Characteristics of patients with eyebrow hypotrichosis who were treated with bimatoprost 0.03% solution *Once daily application of bimatoprost 0.03% solution; however, twice daily application for cases 64-420.

Case	Duration*	Results	Side effects	Reference
1-2	3 months	Satisfied	None reported	[7]
3	4 months	Satisfied	Skin hyperpigmentation	[5]
4-33	4 months	Hair diameter: significant increase noted	Contact dermatitis	[6]
34-53	9 months	Month 2: improvement initially noted Month 6: maximum improvement noted	None reported	[3]
54-63	6 weeks	Marked improvement	None reported	[4]
64-420	7 months	Month 2: improvement initially noted Month 7: maximum improvement noted	Pruritus	[9]
421	8 months	Excellent; complete eyebrow regrowth	None	Current report

Bimatoprost was either applied once daily—like our patient—or twice daily to the eyebrows. Improvement was noted as early as eight weeks; however, maximum improvement was observed to occur between six to eight months [3,9]. Similar to our patient, hair growth was progressive and continued to increase as the patient maintained the topical treatment.

No significant adverse events were recorded. However, one group of investigators observed contact dermatitis in three of 27 patients (11.11%) [6]. Skin hyperpigmentation was also reported in one patient [5]. Pruritus was reported by other researchers in three of 118 patients (2.5%) applying bimatoprost daily and one of 118 patients (0.8%) treating twice a day; however, pruritus was also reported in one of 121 patients (0.8%) who were being treated with the vehicle [9].

## Conclusions

Eyebrow hypotrichosis can be a significant aesthetic concern for the affected individual. Current treatment modalities are limited. Bimatoprost is a novel therapeutic agent that not only treats eyelash hypotrichosis successfully but has also demonstrated efficacy for individuals with hypotrichosis of their eyebrows. Additional studies are warranted to confirm the preliminary observations that we and other researches have made regarding topical bimatoprost for treating eyebrow hypotrichosis. The use of other similar analogs in future studies may potentially expand the armamentarium of treatment options for eyebrow hypotrichosis.
